# Variations in Hospital Expenditure.—I

**Published:** 1889-04-13

**Authors:** 


### Variations in Hospital Expenditure.—I.

Those who are led, either by duty or by interest in the subject, to study hospital reports must be struck by the extraordinary distance that lies between the maximum and minimum of expenditure per occupied bed. Cost per occupied bed does not, of course, mean merely the cost of the board and medicine of the patients who have successfully filled that bed, any more than a man's expenditure means only the amount he spends on food, but the proportionate share belonging to that portion of hospital accommodation represented by a bed in the expenses incurred in keeping up the hospital. The generations of patients being short and fleeting, we must regard the bed as the individual, a corporate, though unconscious entity, having a certain responsibility thrust upon it in matters pecuniary. We set down as the bed's expenditure not only the cost of food, medicine, and dressings provided for its successive occupants; not only such renewal of blankets and bed linen as it may have needed during the year, but its share in the salaries of every official in the hospital, of the coals that cook the food as much as those that keep up the ward fires, of the repairs or enlargements of the premises, and the rent and taxes of the building. It may seem superfluous and even childish to many to set all this down in such exceedingly plain and domestic English, but while a large number of persons look upon "cost per occupied bed" as an equivalent for "patient's board," it is advisable before attempting any comparison between the expenditure of different institutions, to state exactly what is meant by the phrase we shall have to employ in our analysis.

But it is the very variety of items included under this head of "cost per occupied bed" that makes the subject difficult to deal with. Annual reports—meant chiefly for subscribers who presumably know something of the circumstances of the hospital—do not give all the information needful for an enquirer to decide whether or not a particular institution is managed economically or not, and secretaries, when questioned on the subject, seem to have an occult reason for withholding it. Probably their reasons for refusing or neglecting to answer queries may be set down as two very simple ones: "What's the good?" and "It's none of your busines." They have no reason for concealing the facts asked for; but they see none for revealing them. Hence they make any opinion, any absolutely just comparison between kindred institutions difficult in the extreme. For example, if of two institutions having an equal number of beds, one has to pay ground rent, while the other has received its site as a free gift, the first will have a larger annual cost per occupied bed than the second; yet it would be unjust to say, as the figures taken by themselves would lead one to believe, that it is more extravagantly managed than the other.

Again, large establishments cost less in proportion than small ones. In a family of ten, it is commonly said that one more or less doesn't make much difference; it is not so in a family of two. Servants are not increased in exact proportion to newcomers, there are domestic economies of all sorts to be effected by purchasing large quantities instead of small, and in hospitals there is the extra advantage that the larger establishments have the best chance of securing gratuitous services, or even of getting them paid for. Hospitals with medical schools attached to them can have a large choice of house-surgeons for nothing, while provincial infirmaries have to give a salary. All these things cause variations in average expenditure, yet it is difficult to discover the exact amount to which they effect calculations of economy or extravagance based on the cost per occupied bed.

Yet allowing for all these things, it must be admitted that hospital expenses differ in different institutions to an extent not warranted by difference of circumstance. Let us take first metropolitan hospitals, which are more or less similarly situated. The two nearest in size are University College, with 209 beds, and an average daily occupancy of 176, and King's College, with 206 available beds, of which an average of 151 were constantly occupied. The average cost per occupied bed in University College Hospital is £1 10s. 8½d. per week, or £79 16s. 10d. per annum; at King's College Hospital it was £1 18s. 6d. per week, or £100 2s. per annum. (We take the figures from the statistics compiled for the use of the Metropolitan Sunday Fund, 1888.) In these hospitals, of nearly equal size, each having a medical school attached, each free to buy all household and medical necessaries in the same market, each able to obtain the services of officials — from the matron and the secretary and the wardmaid and the porter—at the same salaries, and, to carry out the analogy, each nursed by religious sisters—in these two hospitals, apparently placed in similar circumstances, how is it that the cost per occupied bed differs by over £20 a year? There may be good and sufficient reason for such a remarkable variation, and, indeed, we understand that in the year from the expenditure of which the estimate we quote was compiled extensive alterations in King's College Hospital caused some wards to be closed, and reduced the average number of occupied beds. As during this time the ordinary expenses of management went on—*i.e.*, the salaries of secretary and clerks, printing, collector's commission, and the like—the cost per occupied bed was proportionately raised; for, as we have said, a small institution works, in proportion to its size, more expensively than a large one. Dr. Wace, the chairman of directors at King's College, calculates the number of beds usually occupied at the hospital, when no part of it is shut up, at 169. This will largely modify the cost per occupied bed, which would still, however, reach the tolerably large figure of £92 10s. 3d. per annum.

But King's College has not the largest cost per occupied bed among the metropolitan hospitals. The Middlesex surpasses it, and the Middlesex is a larger hospital, having 310 beds. The average cost there of an occupied bed is £2 2s. per week, or £109 4s. per annum. But there, also, the proportion of occupied beds to available accommodation is unduly small, being only 176 out of a possible 310. If the establishment is conducted in a style proportionate to the available accommodation rather than to that part of it which is used—and to a certain extent it must be—we have at least a partial explanation of the very high rate of expenditure we have quoted. That the London Hospital should be able to treat its patients at the moderate weekly rate of £1 1s. 6¾d. per occupied bed, equivalent to £67 19s. 7d. per annum, is reasonable enough, for it is the largest hospital in the kingdom, and has 644 beds regularly occupied out of a possible 772. It ought to work more cheaply than its poorer brethren, though in saying this we do not wish to imply that its smaller proportionate expenditure is the result of mere accident and not good management.

But in comparing the London with a hospital of almost equal size—the Royal Infirmary at Edinburgh—we find that the English institution does not stand out as a model of economical administration. The cost per occupied bed there is £54 13s. 5d. per annum, or a little less than £1 0s. 7¾d. per week, and we understand in Edinburgh the patients receive full board, which is not the case in all hospitals. We should also add that in the figures we have given for the great Scottish hospital—courteously furnished us by the medical superintendent, Surgeon-General C. H. Fasson —no deduction is made for the out-patient department, while the figures given for the London refer to the in-patients alone. This brings before one another of the difficulties experienced in studying the comparative expenditure of hospitals. Some reports add the cost of the out-patients to the rate per occupied bed, while others carefully deduct it. In the reports of most English hospitals this deduction is made; the reverse is the rule in Scotland.

In point of economy, Edinburgh must, however, yield to Glasgow. The cost per occupied bed at the Royal Infirmary, Glasgow, is £47 3s. 5d. per annum, or about 18s. 1⅝d. per week, and at the Western Infirmary £49 0s. 3d. per annum, or 19s. 10¼d. per week, and at both these institutions the arrangements, both with regard to patients and officials, are remarkably liberal, for it must not be thought that increase of economy means decrease of comfort or efficiency. Those who know the Edinburgh and Glasgow Infirmaries will admit that in general aspect of comfort, as well as in quality of medical attendance and in consideration for the patients' welfare, they are not inferior to any London hospital.

In the English provinces, however, come hospitals managed more cheaply than any of these we have as yet spoken of. For example, Worcester, with an annual rate per occupied bed of £39 14s. 9½d.; the Southern Infirmary at Liverpool, £40, and Manchester Infirmary, £47 4s. 2d. Are we to suppose that this rate, about half the cost per bed of London hospitals, is due to the inferiority of the provincial institutions, or to any disadvantages belonging to a metropolitan situation. The recent improvements in hospitals and their administration are certainly not confined to London, while, on the other hand, as a market, London has unequalled advantages. It certainly looks as if the London hospitals spent more than they need do.

But, as has been already said, it is difficult when one has access only to the ordinary annual report, to say where extravagance comes in, and where an apparently large expenditure is justifiable and necessary. There are certain facts bearing on expenditure which it would be necessary to know before uttering a more definite comment than it may be—it seems that something is—curious. We should like to know :

*1. The rent paid by the hospital (if any).
2. Rates and taxes.
3. Average proportion of beds occupied to available accommodation.
4. Number of nurses in proportion to patients.
5. If any sum is set aside for pensions to past officials.
6. Salaries and wages of the various officials and attendants.
7. Age of the hospital building, with the average annual expenditure on repairs and cleaning.

Some hospital reports do, indeed, give many of these details, but a large majority present only a bald record of income and expenditure, often without explanation of any sort. On such as these all criticism must be imperfect and tentative. To praise or to condemn would alike be unjust when the data on which a judgment must be founded are so insufficient. But we would ask hospital secretaries to look upon their reports as things not meant merely for the indifferent eyes of lay subscribers, but as contributions to hospital literature—pamphlets which convey a certain amount of desired information to their fellow secretaries all over the kingdom, and to all who are interested in hospital administration. If they could be inoculated with such a feeling, they would very soon give the world plenty of material for study of the comparative expenditure of hospitals, and would probably at the same time cause a diminution in the at present remarkable variation in hospital expenditure.

*(To be continued.)*

